# Synthesis and Reactivity of the First Isolated Hydrogen‐Bridged Silanol–Silanolate Anions

**DOI:** 10.1002/anie.201914339

**Published:** 2020-02-11

**Authors:** Robin F. Weitkamp, Beate Neumann, Hans‐Georg Stammler, Berthold Hoge

**Affiliations:** ^1^ Centrum für Molekulare Materialien Fakultät für Chemie Universität Bielefeld Universitätsstraße 25 33615 Bielefeld Germany

**Keywords:** phosphazene, silanolates, silicone, siloxanes, weakly coordinating cations

## Abstract

We report on the first examples of isolated silanol–silanolate anions, obtained by utilizing weakly coordinating phosphazenium counterions. The silanolate anions were synthesized from the recently published phosphazenium hydroxide hydrate salt with siloxanes. The silanol–silanolate anions are postulated intermediates in the hydroxide‐mediated polymerization of aryl and alkyl siloxanes. The silanolate anions are strong nucleophiles because of the weakly coordinating character of the phosphazenium cation, which is perceptible in their activity in polysiloxane depolymerization.

## Graphical abstract

**Silanol–silanolate anions** that are devoid of any contacts to the counterion were synthesized by treatment of siloxane species with a phosphazenium hydroxide hydrate salt. Because of the weakly coordinating cation, the silanolate anions exhibit strong silanol–silanolate hydrogen bonding. The [D_3_OH]^−^ salt was successfully tested in the depolymerization of polydimethylsiloxane with an outstanding activity relative to NaOH or KOH.

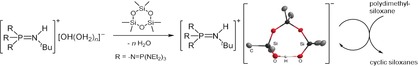

Silicones constitute the passage between organic and inorganic polymers with outstanding chemical and physical properties and are of broad scientific and industrial interest.^1^, ^2^ Linear polydimethylsiloxanes with repeating difunctional (D) units are mostly employed as materials in the modern silicone industry and are largely synthesized by hydrolysis of dimethylchlorosilanes from the Müller–Rochow process (Scheme 1).^2^, ^3^, ^4^, ^5^

**Scheme 1:**
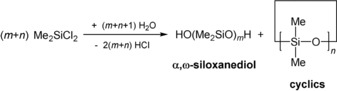
Industrial synthesis of silicones.^4^

The intermediately formed α,ω‐siloxanediols eliminate water under formation of the final silicones or cyclic siloxanes like octamethylcyclotetrasiloxane (D_4_). The latter can be converted into linear polysiloxanes by ionic ring‐opening polymerization reactions.^2^, ^3^, ^4^, ^5^, ^6^

Silanols as well as silanediols are valuable building blocks in synthetical chemistry and exhibit a distinct tendency to form hydrogen‐bridge networks.^7^, ^8^, ^9^, ^10^, ^11^, ^12^, ^13^, ^14^, ^15^ The formation of coordination adducts of silanols with oxygen‐ and nitrogen‐containing bases, which form more readily than the corresponding alcohol adducts,^8^, ^11^ and their utilization for selective guest–host complexation of alcohols and amines emphasize this behavior.^9^, ^12^, ^16^ In the same way, the related α,ω‐siloxanediols, HO[SiR_2_O]_*n*_H, form inter‐ and intramolecular hydrogen bonds that often result in the formation of ring structures (Scheme 2).^13^, ^14^, ^15^

**Scheme 2:**
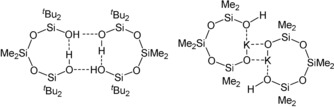
Examples of eight‐membered siloxane rings.^10^, ^14^, ^17^

In the case of the monopotassium silanolate salt (Scheme 2, right), it is noteworthy that the potassium cation interaction with the silanolate as well as the silanol oxygen atom is favored with respect to the formation of an intramolecular hydrogen bond, which correspondingly leads to a ring containing the potassium ion.

Anionic silanolates can be synthesized by cleavage of siloxanes with strong bases.^18^ A subsequent treatment of α,ω‐siloxanediolates with metal halides or alkoxides leads to a broad class of cyclic metallacyclosiloxanes, in which a ring expansion is commonly observed.^19^ Sullivan et al. investigated this ring expansion in more detail and treated salts of the tetraphenyldisiloxanediolate anion, [O(SiPh_2_O)_2_]^2−^, with metal halides under several reaction conditions, which resulted in the formation of six‐ and eight‐membered metallacyclosiloxanes.^20^ Although the reaction mechanism has not been completely elucidated, the formation of eight‐membered siloxane rings via repeated rearrangements of Si−O bonds seems thermodynamically favorable.

Regarding the chemical robustness of silicones in nature, their multi‐ton production causes serious waste disposal problems. The degradation of siloxanes under formation of cyclic derivatives is of general interest because it forms the basis of a possible recycling of widely used silicone plastics.

The depolymerization of silicones can be achieved under acidic, basic, and fluorinating conditions, leading to low‐molecular cyclic siloxanes or organosilyl fluorides, which can be converted into new silicone plastics afterwards.^21^, ^22^ Known processes for the depolymerization of silicones utilize metal hydroxides such as KOH but require elevated temperatures. A pronounced interaction between the potassium cation and the silanolate anion and therefore a reduced nucleophilicity may be considered as a reasonable explanation for the relatively low conversion rate.

Recently, we reported the synthesis of the phosphazenium hydroxide salt [**1**H][OH(OH_2_)_*n*_] (**2**; Scheme 3), which exhibits pronounced reactivity due to the weakly coordinating character of the phosphazenium ion.^23^

**Scheme 3:**
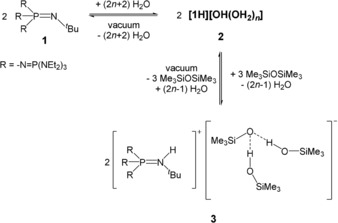
Equilibrium reaction of phosphazene **1**, hydroxide salt **2**, and silanol–silanolate salt **3**.

In the following work, the reaction of the hydroxide salt **2** with siloxanes and polysiloxanes was in the focus of our interest in order to examine the nature of silanolate anions that do not show direct contact to the counterion and to investigate their application as a depolymerization catalyst for polysiloxanes.

The reactions of **2** with siloxane species were carried out in *n*‐hexane as a nonpolar solvent, which was chosen because of the fast decomposition of **1** and **2** in H‐acidic, even C−H‐acidic, solvents and the beneficial precipitation of ionic products.^24^

A mixture of equimolar quantities of phosphazene **1** and hexamethyldisiloxane showed no reaction. The hydroxide salt **2** was generated by the addition of one equivalent of water to the mixture. Hydroxide salt **2** precipitates as an amorphous colorless solid, which is rapidly consumed to yield a slightly yellowish second phase. The obtained crystalline product **3**, which was isolated from the cooled reaction mixture and analyzed by single‐crystal X‐ray diffraction (Figure 1), features a silanolate anion that is bonded to two silanol molecules.^25^ To increase the yield of **3**, the run was repeated accordingly with the appropriate stoichiometry of the reactants (Scheme 3).^24^

**Figure 1:**
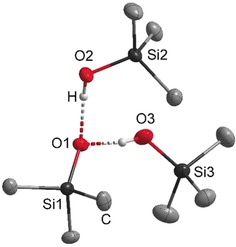
Molecular structure of the silanolate anion in **3**. The phosphazenium cation and minor occupied disordered atoms are not shown. Thermal ellipsoids set at 50 % probability. The hydrogen atoms of the methyl groups are omitted for clarity. Selected bond lengths [pm] and angles [°]: O1–O2 258.2(1), O1–O3 252.4(5), O1–Si1 159.9(1), O2–Si2 162.4(1), O3–Si3 161.8(3); Si1‐O1‐O2 118.0(1), Si1‐O1‐O3 124.6(1), O2‐O1‐O3 102.7(2).^25^

Compound **3** is the first example of an isolated silanol–silanolate anion that is not in direct contact with any counterion. O3 is disordered at two positions with a ratio of 79:21 and shows the shortest distances to C37 with 331.5(4) and 329.0(1) ppm; both distances are longer than the sum of the van der Waals radii. The donor hydrogen atom is disordered as well, and bonded to O1 or O3. Both positions were refined isotropically, but restrained to have the same O−H distances.

Compared to the O–O distances of the hydrogen bridges in the triphenylsilanol pyrrolidine complex of Strohmann and co‐workers (249.1, 270.1, 283.3 pm),^12^ the hydrogen bridges in the anion of salt **3** exhibit an O1–O2 distance of 258.2(1) pm and an O1–O3 distance of 252.4(5) pm.

As already stated for the strongly basic compounds **1** and **2**, silanolate salt **3** undergoes rapid decomposition in H‐ and C−H‐acidic solvents and requires handling in chlorobenzene solution. A ^31^P and ^29^Si{^1^H} NMR spectroscopic investigation of the dissolved product **3** indicated the presence of the protonated phosphazene **[1H]^+^** and also a reformation of Me_3_SiOSiMe_3_, which points to the equilibrium reaction shown in Scheme 3. The signal in the ^29^Si{^1^H} NMR spectrum at *δ*=−8.6 ppm was attributed to the silicon atoms of the silanolate anion in **3**. As observed for the hydroxide hydrate salt **2**, silanolate salt **3** decomposes in vacuum by the deprotonation of its cation **[1H]^+^** and liberation of silanol and disiloxane.^23^ This observation is further evidence for the equilibrium reaction between hydroxide **2** and the silanolate anion in **3** (Scheme 3). This situation hampers a reliable elemental analysis of salt **3**. By this route, the synthesis of a “naked” trimethylsilanolate anion in the presence of the phosphazenium ion **[1H]^+^** is not possible.

In the following, phosphazene **1** was combined with a small excess of hexamethylcyclotrisiloxane (D_3_). After the addition of water, the reaction with the in situ generated hydroxide produced a second phase. In the upper phase, a mixture of cyclic compounds, mainly D_4_ and D_5_ and traces of D_3_, were detected by ^1^H–^29^Si HMBC NMR spectroscopy. This result clearly underlines the existence of a fast equilibrium between cyclic species, whose interconversion is catalyzed by **2**. The initial ring‐opening reaction is presented in Scheme 4. ^31^P NMR spectroscopic analysis confirmed that there are no phosphorus species in the upper phase.

**Scheme 4:**
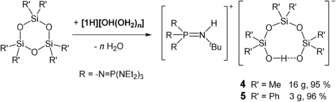
Reaction of cyclotrisiloxanes with in situ generated phosphazenium hydroxide.

Upon cooling the reaction mixture to −28 °C, colorless crystals of silanolate salt **4** were obtained in an excellent yield of 95 %. X‐ray crystallographic analysis^25^ revealed a cyclic silanol–silanolate anion of the type [D_3_OH]^−^ in **4**, which forms an intramolecular hydrogen bridge in the solid state (Figure 2). An elemental analysis underlined the selective formation of compound **4** in high yields (Scheme 4; calcd: C 49.04, H 10.65, N 16.16, P 11.00, Si 7.48; found: C 48.61, H 10.64, N 15.89, P 10.98, Si 7.59).

**Figure 2:**
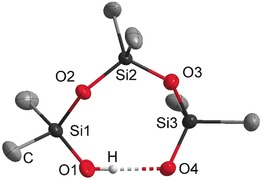
Molecular structure of the [D_3_OH]^−^ anion in **4**. The phosphazenium cation is not shown. Thermal ellipsoids set at 50 % probability. The donor hydrogen atom is disordered in a ratio of 1:1, bonded to O1 or O4, but only one is shown. The hydrogen atoms of the methyl groups are omitted for clarity. Selected bond lengths [pm] and angles [°]: O1–O4 242.8(2), O1–Si1 157.9(2), O4–Si3 158.9(1), O2–Si2 161.8(1), O2–Si1 165.8(1); O4‐O1‐Si1 124.4(1), O1‐O4‐Si3 113.2(1), O2‐Si2‐O3 112.7(1).^25^

The oxygen atoms are well separated from the phosphazenium cation. The shortest distance of 304.4(2) pm is observed between O1 and C31. The disordered donor hydrogen atom was refined isotropically at two positions with a ratio of 1:1; both were restrained to have the same distances to oxygen atoms O1 and O4, respectively.

Based on potentiometric titrations of siloxanes, the thermodynamically favored eight‐membered‐ring structure was already proposed by Baney and Atkari in 1967.^26^ The O1–O4 distance was determined to be 242.8(2) pm and points to strong hydrogen bonding.^27^ The O4−Si3 and O1−Si1 bond lengths range from 157.8(2) to 158.9(1) pm and are slightly shortened in comparison to the eight‐membered siloxane ring D_4_ (Si‐O distances from 164 to 166 pm).^28^

The O‐H‐O vibration mode of **4** could not be determined in the IR analysis. The ^1^H NMR spectrum in C_6_D_6_ displayed a broad singlet at *δ*=14.0 ppm, which was assigned to the proton involved in the hydrogen bridging. In the ^29^Si{^1^H} NMR spectrum of **4** the signal for the silicon atoms adjacent to the hydrogen bridge was observed at *δ*=−23.9 ppm.

Compound **4** is also accessible from the treatment of D_4_ or D_5_ with the phosphazenium hydroxide, as evident by NMR and X‐ray analysis. The reaction of **2** with equimolar quantities of D_5_ afforded compound **4** in a 85 % yield. A plausible reaction pathway mirrors a series of equilibria and confirms the high thermodynamic stability of the hydrogen‐bridged eight‐membered ring.

Silanolate salt **4** begins to decompose slowly at 90 °C in vacuo, with fast decomposition above 100 °C. The volatile products were identified by GC‐MS analysis as water and cyclic siloxanes, with D_4_ as the main component. A ^31^P NMR spectroscopic investigation of the residue revealed the free phosphazene **1**, and the decomposition route is thus similar to that of hydroxide hydrate **2**.

The reaction of in situ generated phosphazenium hydroxide with hexaphenylcyclotrisiloxane in diethyl ether as a solvent results in the clean formation of the analogous [D^Ph2^_3_OH]^−^ anion in salt **5**, which was isolated in excellent yield (>96 %; Scheme 4). Single crystals of **5** were obtained from a cooled reaction mixture (−28 °C) and were subjected to X‐ray crystallography (Figure 3).^25^

**Figure 3:**
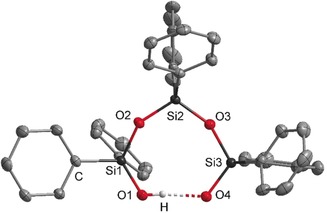
Molecular structure of the [D^Ph2^_3_OH]^−^ anion in **5**. The phosphazenium cation is not shown. Thermal ellipsoids set at 50 % probability. The donor hydrogen atom is disordered in a ratio of 1:1, bonded to O1 or O4, but only one is shown. The hydrogen atoms of the phenyl groups are omitted for clarity. Selected bond lengths [pm] and angles [°]: O1–O4 242.9(2), O1–Si1 158.4(1), O4–Si3 157.8(1), O2–Si1 164.9(1), O2–Si2 162.1(1); O1‐O4‐Si3 125.1(1), O4‐O1‐Si1 119.0(1), O2‐Si2‐O3 112.3(1).^25^

The disordered donor hydrogen atom was refined isotropically at two positions with a ratio of 1:1; both were restrained to have the same distances to the oxygen atoms O1 and O4, respectively. The O1–O4 distance of 242.9(2) ppm shows the same value as in **4**. Analogously to compound **4**, the O‐H‐O vibration mode of salt **5** could not be determined by IR analysis.

Because of the weakly coordinating nature of the phosphazenium counterion, the hydroxide hydrate anion in **2**, as well as the silanolate anions in **3**, **4**, and **5** exhibit increased nucleophilicity. This was exemplary shown for silanolate salt **4** in the depolymerization reaction of a polydimethylsiloxane with terminal trimethylsilyl groups (Scheme 5).

**Scheme 5:**
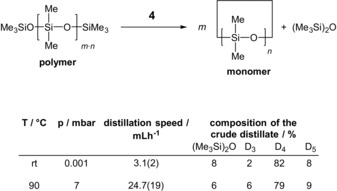
Depolymerization of trimethylsilyl‐end‐blocked polydimethylsiloxanes employing silanolate salt **4** (see also Tables S1–S3).^24^

The increased nucleophilicity of the silanolate oxygen atom is accompanied by a fast equilibrium reaction, which is distinguished by back‐biting and end‐biting processes.^22^

The equilibrium activity can be determined by the temporal quantity of cyclic siloxane species, which are removed from the siloxane mixture by distillation. The silanolate salt **4** was applied in a concentration of 0.1 mol %. Salt **4** showed poor solubility at room temperature and remained present as fine colorless particles. Nevertheless, cyclic siloxane species and hexamethyldisiloxane were formed and entirely removed in vacuum (0.001 mbar) with an average rate of 3.1(2) mL h^−1^.^24^ After completion of this process, catalyst **4** was regained as a colorless solid devoid of any traces of decomposition. The obtained distillate consisted mainly of D_4_ (82 %) as well as D_5_ (8 %) and traces of D_3_ (2 %; see the Supporting Information, Table S2).^24^ Trimethylsiloxane species (8 %) were isolated as well.

As industrial processes are preferably run at higher temperatures and higher pressures, we also mimicked these conditions by applying a membrane pump vacuum (7 mbar) and a temperature of 90 °C. Under these conditions, a clear solution of **4** in silicone oil was formed, whereby the averaged distillation rate of volatiles was determined to be 24.7(19) mL h^−1^. The average composition of the distillate does not significantly differ from that of the run at room temperature (Scheme 5). In comparison to the results in sodium (0 mL h^−1^) or potassium hydroxide (<1 mL h^−1^) mediated depolymerizations with concentrations of 11 mol % carried out at 90 °C, compound **4** showed a significantly enhanced equilibrium activity (Table S1 in the Supporting Information).^24^

In conclusion, we have reported on the first three silanol–silanolate anions in the condensed phase, which were synthesized by the reaction of the in situ generated hydroxide hydrate anion [OH(OH_2_)_*n*_]^−^ in **2** in the presence of the weakly coordinating phosphazenium cation **[1H]^+^**. The silanolate anions are not in contact with the counterion, and the [D_3_OH]^−^ salt **4** and the [D^Ph2^_3_OH]^−^ salt **5** show only intramolecular hydrogen bonding. Similar to the hydroxide hydrate salt **2**, trimethylsilanolate salt **3** decomposes in vacuum. An NMR spectroscopic investigation provided evidence for an equilibrium reaction of hydroxide salt **2** and silanolate salt **3**. The salts [**1**H][D_3_OH] (**4**) and [**1**H][D^Ph2^_3_OH] (**5**) were synthesized in excellent yields of over 95 % and structurally characterized.

The increased nucleophilicity of the silanolate anion in salt **4** was used to perform a fast solvent‐free depolymerization of polydimethylsiloxanes into cyclic siloxanes. Under identical reaction conditions, the catalytic activity of silanolate **4** was significantly higher than that of sodium and potassium hydroxide.

## Conflict of interest

The authors declare no conflict of interest.

## Supporting information

As a service to our authors and readers, this journal provides supporting information supplied by the authors. Such materials are peer reviewed and may be re‐organized for online delivery, but are not copy‐edited or typeset. Technical support issues arising from supporting information (other than missing files) should be addressed to the authors.

Supplementary
