# Supplementary material for: Synthesis and Reactivity of the First Isolated Hydrogen‐Bridged Silanol–Silanolate Anions
Source: Angew Chem Int Ed Engl. 2020 Feb 11;59(14):5494–9. doi: 10.1002/anie.201914339 (PMC7154667; doi:10.1002/anie.201914339)
Supplement: Supplementary file 1 — Supplementary [file ANIE-59-5494-s001.pdf]

Angewandte Chemie
Eine Zeitschrift der Gesellschaft Deutscher Chemiker

Supporting Information

Synthesis and Reactivity of the First Isolated Hydrogen-Bridged Silanol–Silanolate Anions

*Robin F. Weitkamp, Beate Neumann, Hans-Georg Stammler, and Berthold Hoge**

anie_201914339_sm_miscellaneous_information.pdf

1. Experimental Section

1.1 General Part

All chemicals were obtained from commercial sources and used without further purification. Standard high-vacuum techniques were employed throughout all experiments. Non-volatile compounds were handled in a dry N_2 atmosphere using Schlenk techniques.

1.2 Analysis Methods

1.2.1 NMR Spectroscopy

NMR spectra were recorded on a Bruker Model Avance III 300 spectrometer (^{1}H 300.13 MHz; ^{13}C 75.47 MHz; ^{29}Si 59.63 MHz; ^{31}P 121.49 MHz) or on a Bruker Avance III 500 HD spectrometer (^{1}H 500.20 MHz; ^{13}C 125.79 MHz; ^{29}Si 99.38 MHz; ^{31}P 202.48 MHz). Positive shifts are downfield from the external standards TMS (^{1}H, ^{13}C, ^{29}Si) and H_3PO_4 (^{31}P). The NMR spectra were recorded in the indicated deuterated solvent or in relation to acetone-d_6 filled capillaries.

1.2.2 IR Spectroscopy

IR spectra were recorded on an ALPHA-FT-IR spectrometer (Bruker) using an ATR unit with a diamond crystal for liquids and solids.

1.2.3 Elemental Analyses

Elemental analyses were performed by Mikroanalytisches Laboratorium Kolbe (Oberhausen, Germany). The elemental analysis of [1H][$D^{Ph2}{}_3$OH] (**5**) was accomplished in the element-analytical laboratory of the Universität Bielefeld using the EURO EA Element Analyzer 2010 (HEKAtech GmbH).

1.2.4 Melting Point

Melting points were measured on a Mettler Toledo Mp70 Melting Point System.

1.3 Syntheses

1.3.1 Generation of [1H][$Me_3SiO(HOSiMe_3)_2$] (3)

Phosphazene **1** (2.50 g, 2.82 mmol) and hexamethyldisiloxane (0.69 g, 4.24 mmol) are diluted in 10 mL of *n*-hexane, and water (80 mg, 4.44 mmol) is added to form a two-phase system. The reaction mixture is stirred for 9 hours and then cooled in a fridge overnight. The supernatant is removed via a syringe. By adding *n*-pentane (10 mL) and cooling of the resulting emulsion in a fridge, small yellowish crystals were obtained.

^1H NMR (C_6H_5Cl, rt): δ [ppm] = -0.3 (s, hexamethyldisiloxane), 0.1 (s, Me_3SiO/OH), 0.6 (t, $^3J_{HH}$ = 7 Hz, 54 H, CH_3), 0.8 (s, 9 H, $C(CH_3)_3$), 1.5 (d, $^2J_{PH}$ = 8 Hz, 1 H, NH), 2.5 (dq, $^3J_{PH}$ = 10 Hz, $^3J_{HH}$ = 7 Hz, 36 H, CH_2), 8.5 (s, 1 H, OH).
$^{13}C\{^1H\}$ NMR (C_6H_5Cl, rt): δ [ppm] = 4.1 (s, Me_3SiO-/OH), 12.8 (d, $^3J_{PC}$ = 4 Hz, CH_3), 30.9 (d, $^3J_{PC}$ = 5 Hz, C(**C**H_3)$_3$), 38.5 (d, $^2J_{PC}$ = 6 Hz, CH_2), 50.0 (d, $^2J_{PC}$ = 4 Hz, **C**(CH_3)$_3$).
$^{29}Si\{^1H\}$IG NMR (C_6H_5Cl, rt): δ [ppm] = -8.6 (Me_3SiO/OH).
^{31}P NMR (C_6H_5Cl, rt): δ [ppm] = -34.4 (q, d, $^2J_{PP}$ = 70 Hz, $^2J_{PH}$ = 8 Hz, 1 P, P=NH), 6.8 (d, tridec, $^2J_{PP}$ = 70 Hz, $^3J_{PH}$ = 10 Hz, 3 P, $(Et_2N)_3P$).
IR (ATR): $\tilde{\nu}$ [cm^{-1}] = 3383 (vw), 2966 (w), 2935 (vw), 2872 (vw), 1641 (vw), 1463 (vw), 1378 (w), 1351 (w), 1270 (m), 1202 (m), 1174 (vs), 1107 (w), 1054 (w), 1017 (vs), 940 (s), 844 (w), 784 (s), 740 (w), 700 (m), 614 (w), 508 (s), 440 (m).

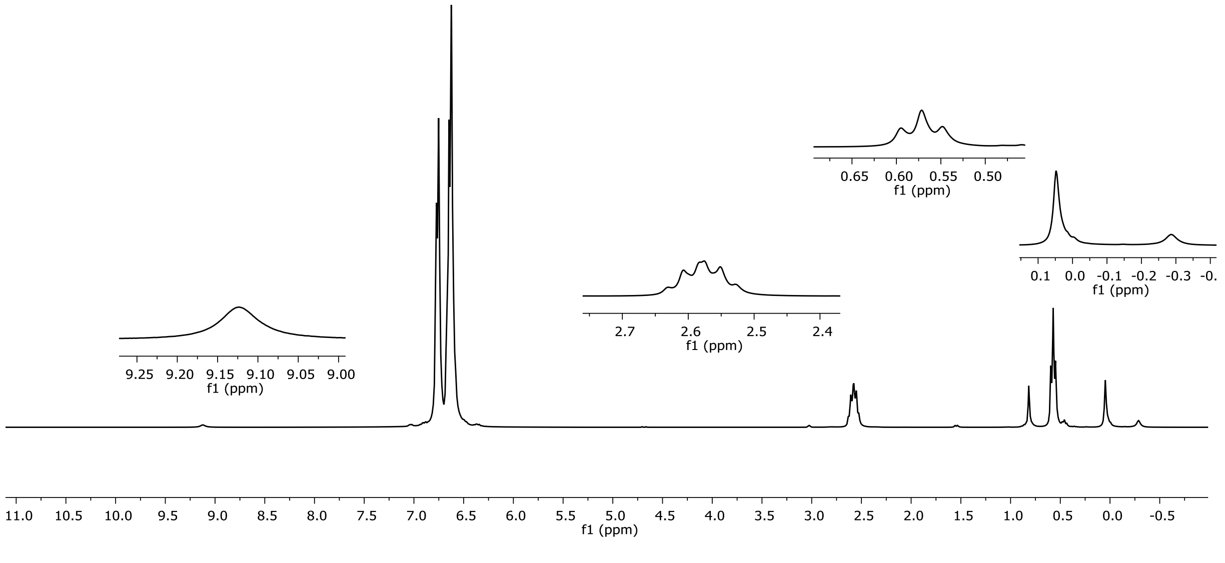

Figure 1. ^1H NMR spectrum of **3** in chlorobenzene (lock with acetone-d_6 in a capillary) (300 MHz).

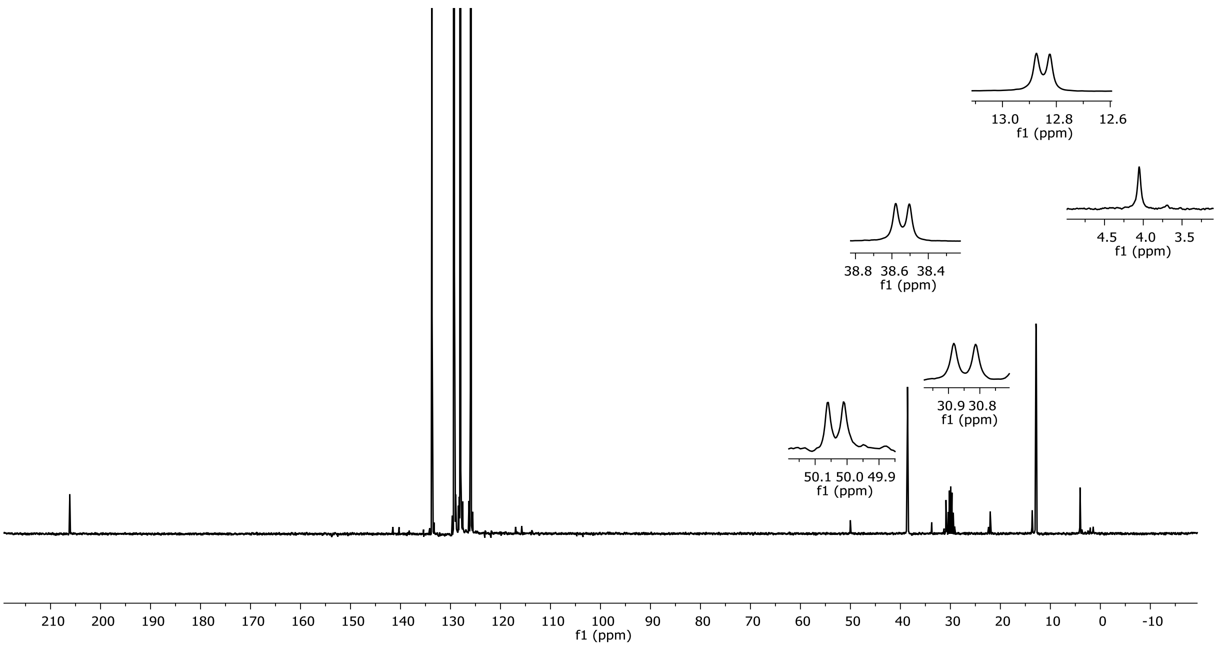

Figure 2. $^{13}C\{^{1}H\}$ NMR spectrum of **3** in chlorobenzene (lock with acetone-d_6 in a capillary) (300 MHz).

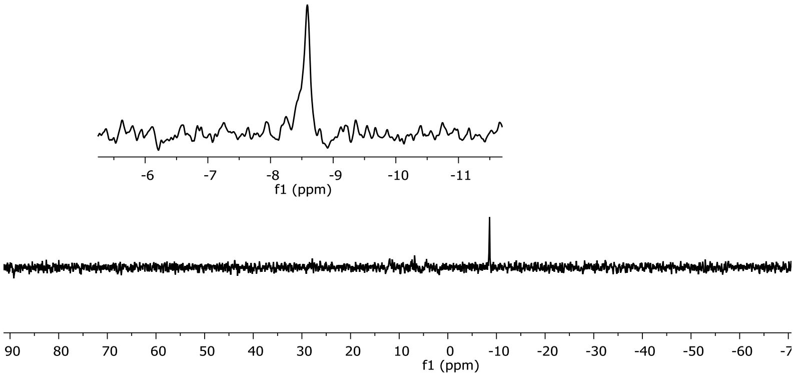

Figure 3. $^{29}Si\{^{1}H\}$IG NMR spectrum of **3** in chlorobenzene (lock with acetone-d_6 in a capillary) (300 MHz).

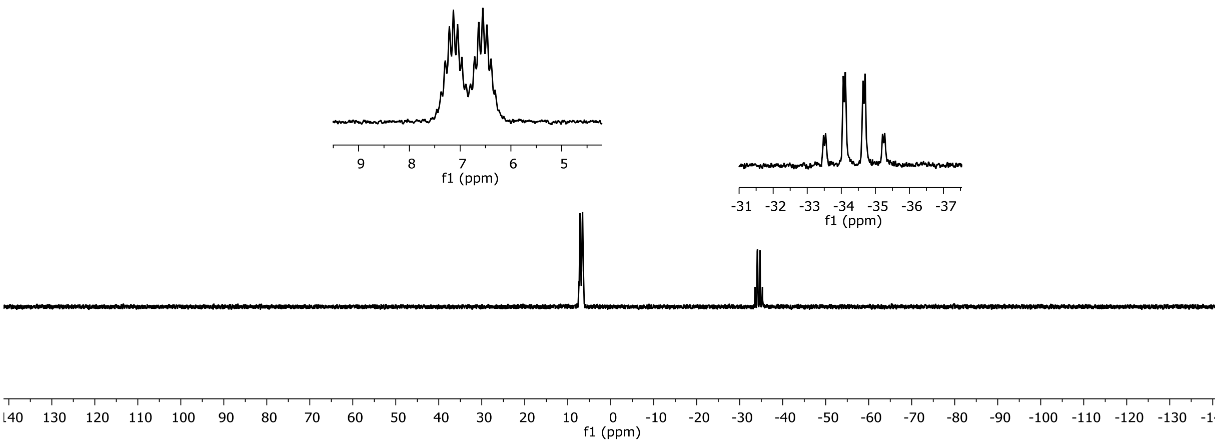

Figure 4. ^{31}P NMR spectrum of **3** in chlorobenzene (lock with acetone-d_6 in a capillary) (300 MHz).

1.3.2 Synthesis of [1H][D_3OH] (4)

Phosphazene **1** (13.69 g, 15.45 mmol) is dissolved in 45 mL of *n*-hexane before hexamethylcyclotrisiloxane (3.44 g, 15.45 mmol) is added. After addition of water (0.29 g, 15.45 mmol) two phases separated. After stirring at room temperature overnight, a pale yellow solid precipitates. The supernatant solution is removed via a syringe and the solid is dried in high vacuum (10^{-3} mbar). The product (16.46 g, 14.61 mmol, 95 % based on **3**) is obtained as a colorless crystalline solid (m.p. 99-101 °C).

^{1}H NMR (C_6D_6, rt): δ [ppm] = 0.6 (s, 7 H, SiO(H_3C)$_2$SiOSi), 0.7 (s, 11 H, (H_3C)$_2$SiO), 1.0 (t, $^3J_{HH}$ = 7 Hz, 54 H, CH_3), 1.3 (s, 9 H, C(CH_3)$_3$), 2.1 (d, $^2J_{PH}$ = 8 Hz, 1 H, NH), 3.0 (d, q, $^3J_{PH}$ = 10 Hz, $^3J_{HH}$ = 7 Hz, 36 H, CH_2), 14.0 (s, OH).
^{13}C{^{1}H} NMR (C_6D_6, rt): δ [ppm] = 2.3 (s, SiO(H_3C)$_2$SiOSi), 3.6 (s, (H_3C)$_2$SiOH-OSi(CH_3)$_2$), 13.4 (d, $^3J_{PC}$ = 4 Hz, CH_3), 31.3 (d, $^3J_{PC}$ = 5 Hz, C(**C**H_3)$_3$), 39.0 (d, $^2J_{PC}$ = 6 Hz, CH_2), 50.4 (d, $^2J_{PC}$ = 4 Hz, **C**(CH_3)$_3$).
^{29}Si{^{1}H}dept30 NMR (C_6D_6, rt): δ [ppm] = -24.1 (s, SiO(H_3C)$_2$**Si**OSi), -23.9 (s, (H_3C)$_2$SiOH-OSi(CH_3)$_2$).
^{31}P NMR (C_6D_6, rt): δ [ppm] = -33.7 (q, d, $^2J_{PP}$ = 70 Hz, $^2J_{PH}$ = 8 Hz, 1 P, P=NH), 7.6 (d, tridec, $^2J_{PP}$ = 70 Hz, $^3J_{PH}$ = 10 Hz, 3 P, (Et_2N)$_3$P).
IR (ATR): $\tilde{\nu}$ [cm^{-1}] = 2966 (vw), 2872 (vw), 1627 (vw, vbr), 1464 (vw), 1379 (w), 1351 (w), 1273 (m, br), 1247 (m), 1202 (m), 1175 (s), 1053 (w), 1016 (vs), 940 (vs), 847 (w), 784 (vs), 700 (m), 614 (w), 508 (m), 440 (m).
elemental analysis of $C_{46}H_{119}N_{13}O_4P_4Si_3$ (M = 1126.7 g/mol): calcd.: C 49.04, H 10.65, N 16.16, P 11.00, Si 7.48; found: C 48.61, H 10.64, N 15.89, P 10.98, Si 7.59.

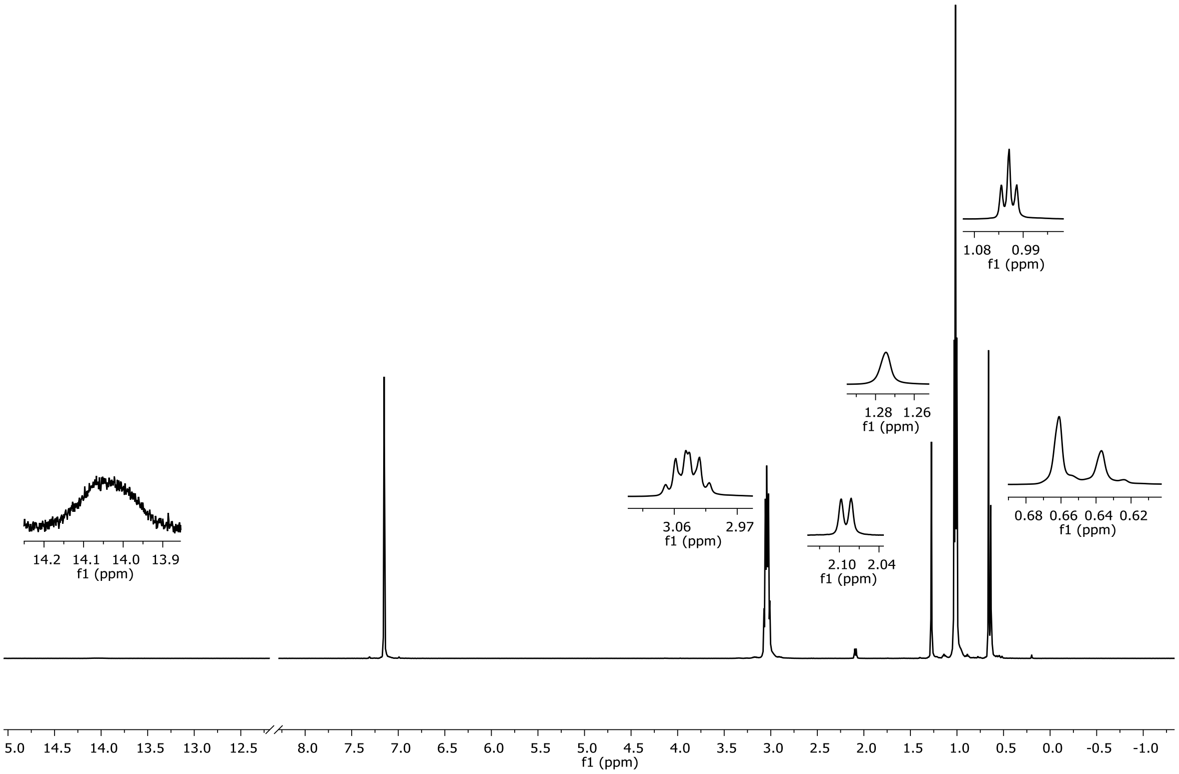

Figure 5. ^{1}H NMR spectrum of **4** in benzene-d$_6$ (500 MHz).

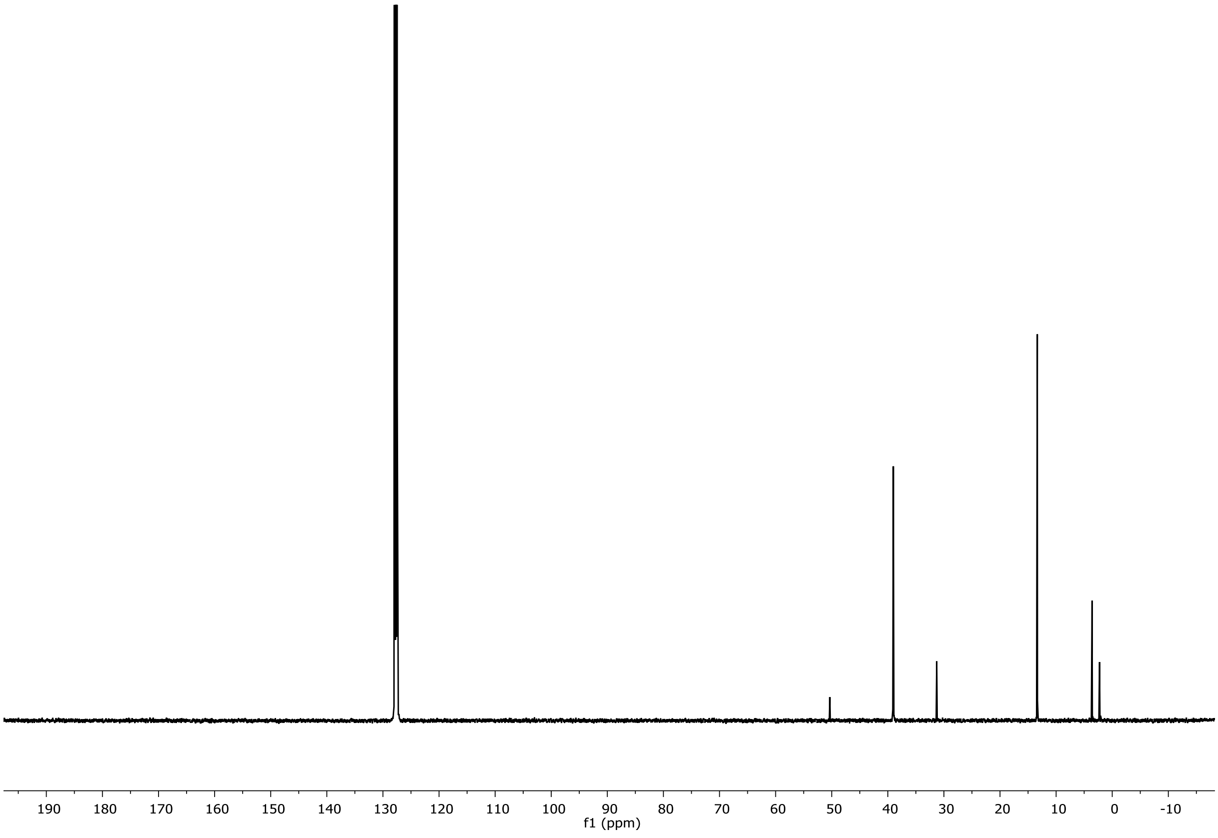

Figure 6. $^{13}C\{^{1}H\}$ NMR spectrum of **4** in benzene-d_6 (500 MHz).

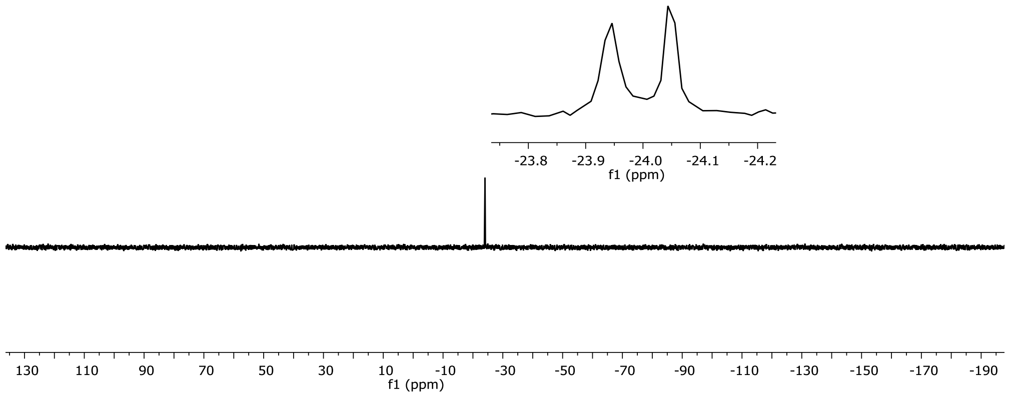

Figure 7. $^{29}Si\{^{1}H\}$dept30 NMR spectrum of **4** in benzene-d_6 (500 MHz).

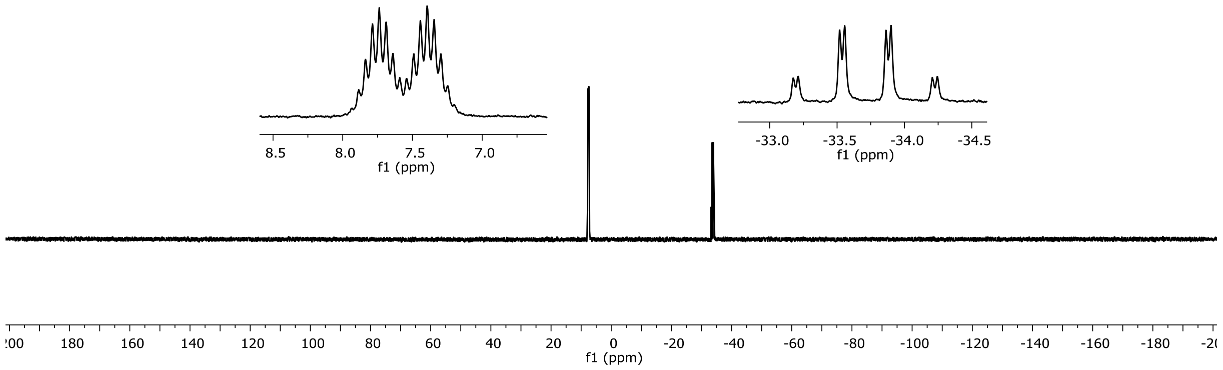

Figure 8. ^{31}P NMR spectrum of **4** in benzene-d_6 (500 MHz).

1.3.3 Synthesis of [1H][D$^{Ph2}{}_3$OH] (5)

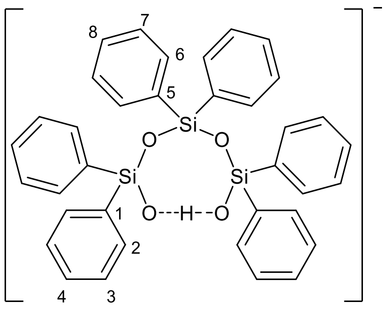

To a solution of **1** (1.69 g, 1.90 mmol) in 10 mL of diethylether hexaphenylcyclotrisiloxane (1.13 g, 1.90 mmol) and water (46 mg, 2.55 mmol) are added. After one hour of stirring, the solution was kept at -28 °C overnight. The solvent is removed and the product (2.74 g, 1.82 mmol, 96 % based on **3**) is isolated as a colorless crystalline solid (m.p. 113-115 °C).

^{1}H NMR (C_6D_6, rt): δ [ppm] = 0.9 (t, $^{3}J_{HH}$ = 7 Hz, 54 H, CH_3), 1.2 (s, 9 H, $C(CH_3)_3$), 2.0 (d, $^{2}J_{PH}$ = 8 Hz, 1 H, NH), 3.0 (d,q, $^{3}J_{PH}$ = 10 Hz, $^{3}J_{HH}$ = 7 Hz, 36 H, CH_2), 7.1 (m), 7.3 (m), 8.1 (m), 8.3 (m), 16.1 (s, br, SiOH).

^{13}C{^{1}H} NMR (C_6D_6, rt): δ [ppm] = 13.3 (d, $^{3}J_{PC}$ = 4 Hz, CH_3), 31.2 (d, $^{3}J_{PC}$ = 5 Hz, C(**C**H_3)$_3$), 38.9 (d, $^{2}J_{PC}$ = 5 Hz, CH_2), 50.3 (d, $^{2}J_{PC}$ = 4 Hz, **C**$(CH_3)_3$), 126.7 (s, C3), 127.0 (s, C7), 128.2 (s, C4/C8), 128.3 (s, C4/C8), 135.2 (s, C6), 135.5 (s, C2), 139.3 (s, C5), 143.8 (s, C1).

^{29}Si{^{1}H}IG NMR (C_6D_6, rt): δ [ppm] = -46.1 (s, 1 Si, SiOPh_2**Si**OSi), -43.8 (s, 2 Si, Ph_2**Si**OH-O**Si**Ph_2).

^{31}P NMR (C_6D_6, rt): δ [ppm] = -34.2 (q, d, $^{2}J_{PP}$ = 70 Hz, $^{2}J_{PH}$ = 8 Hz, 1 P, P=NH), 6.9 (d, tridec, $^{2}J_{PP}$ = 70 Hz, $^{3}J_{PH}$ = 10 Hz, 3 P, $(Et_2N)_3P$).

IR (ATR): $\tilde{\nu}$ [cm^{-1}] = 2969 (vw), 2926 (vw), 2863 (vw), 1464 (vw), 1425 (w), 1379 (w), 1348 (w), 1288 (w), 1264 (m), 1226 (w), 1202 (m), 1173 (s), 1114 (m), 1101 (m), 1074 (m), 1058 (m), 1019 (s), 942 (m), 919 (w), 845 (w), 793 (m), 740 (m), 697 (vs), 657 (m), 613 (m), 590 (w), 579 (w), 523 (vs), 493 (vs), 462 (s), 442 (vs), 434 (vs), 406 (vs).

elemental analysis of $C_{76}H_{131}N_{13}O_4P_4Si_3$ (M = 1499.1 g/mol): calcd.: C 60.89, H 8.81, N 12.15; found: C 60.23, H 8.93, N 12.06.

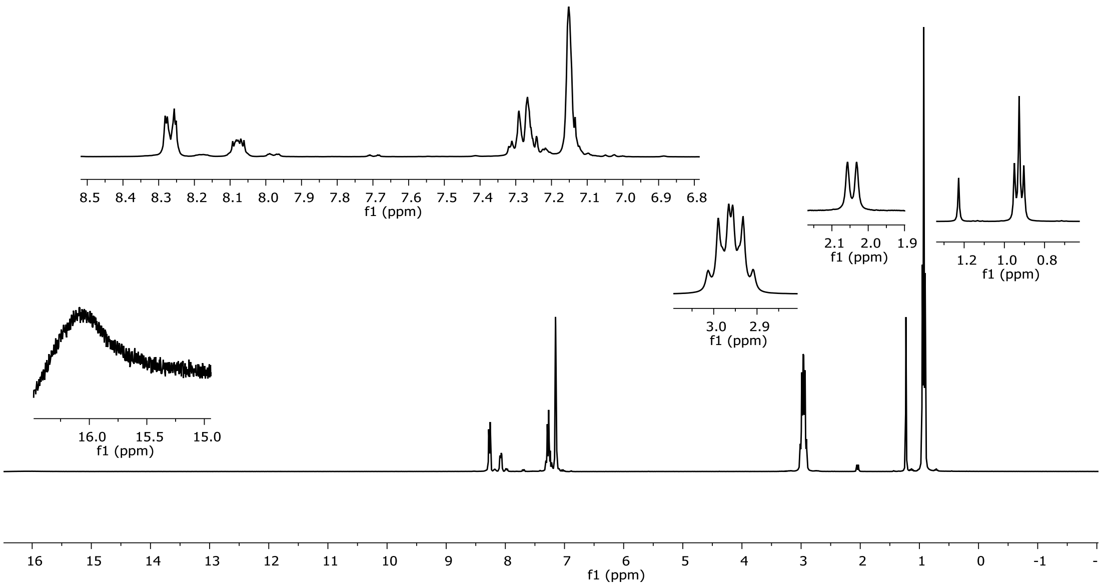

Figure 9. ^{1}H NMR spectrum of **5** in benzene-d$_6$ (300 MHz).

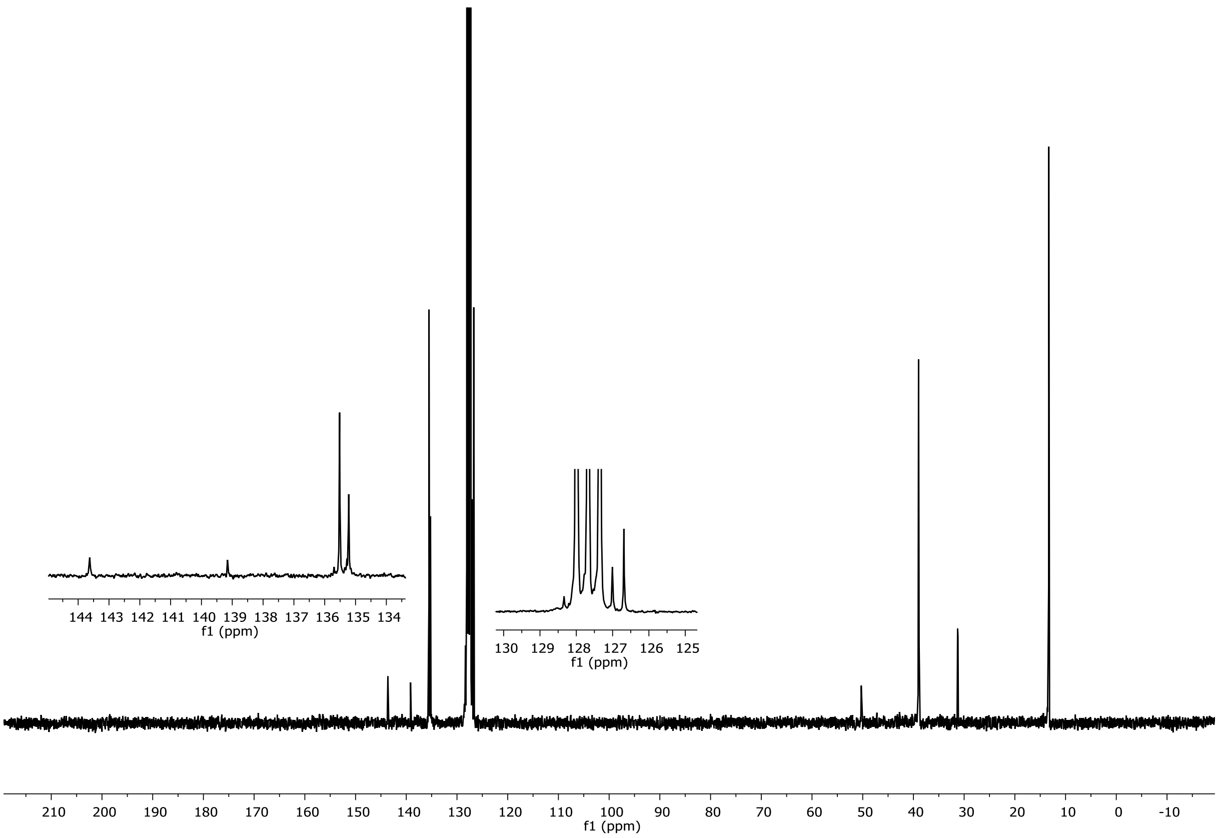

Figure 10. $^{13}C\{^{1}H\}$ NMR spectrum of **5** in benzene-d$_6$ (300 MHz).

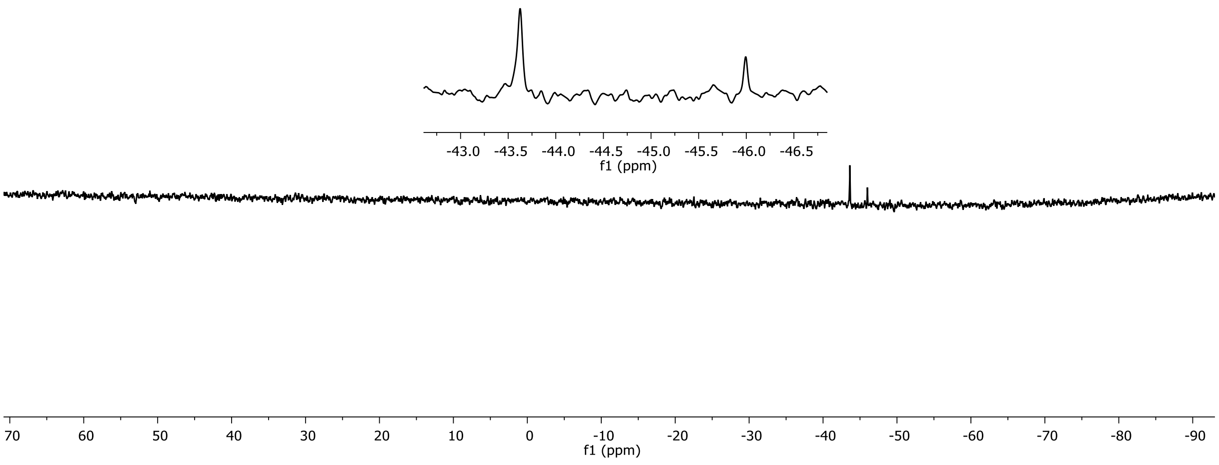

Figure 11. $^{29}Si\{^{1}H\}$IG NMR spectrum of **5** in benzene-d_6 (300 MHz).

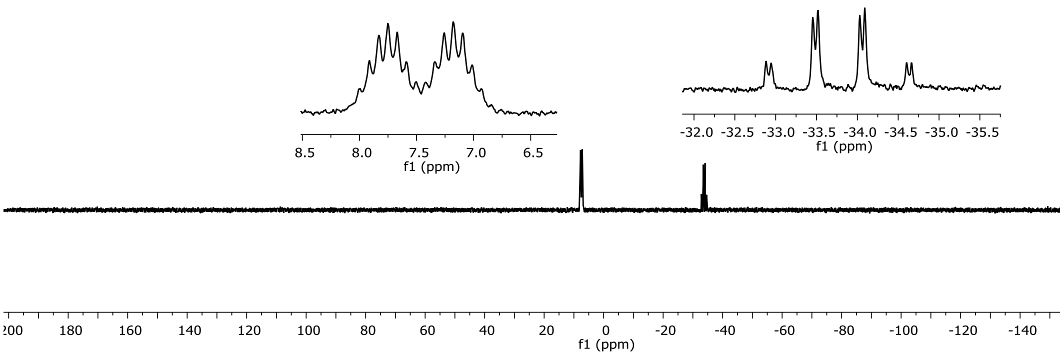

Figure 12. ^{31}P NMR spectrum of **5** in benzene-d_6 (300 MHz).

1.4 Depolymerization reactions of polydimethylsiloxane

In a 250 mL Young flask pure silicon oil (110 g, 1.48 mol, polydimethylsiloxane, Roth Siliconöl M 200, Art.-Nr. 4030.1) is charged with catalyst **4** (1.68 g, 1.49 mmol, 0.1 mol%). Under stirring at room temperature the vessel is evacuated to 0.001 mbar. Thereby generated cyclic products are collected in a cold trap. The distillation speed was determined by weighing the amount of the crude distillate in dependence of time (Figure 13). After complete removal of volatile compounds, catalyst **4** remains as residue.

Afterwards the flask with catalyst **4** is refilled with silicone oil. The temperature is raised to 90 °C and the pressure is raised to 7 mbar. The cooling trap is exchanged by a graduated flask and the distillation speed (Figure 13) is volumetrically determined, by using the first distillation flashover as starting point. The catalyst was reused by adding further amounts of silicone oil after complete turnover. A small amount of silicone oil is kept in the flask to prevent the slow thermolysis of pure catalyst **4**, which is not observed at room temperature. The results are graphically shown in Figure 13.

The collected data were confirmed by an additional depolymerization run at room temperature (0.001 mbar) and two additional runs at 90 °C (7 mbar) with a given amount of polydimethylsiloxane and load of catalyst **4** (results in Tables 1 - 3).

For the comparison with alkali hydroxides as a catalyst, potassium and sodium hydroxide were used as a catalyst for the depolymerization reactions, as shown in Table 1, using identical conditions (90 °C, 7 mbar) for the comparison with catalyst **4**.

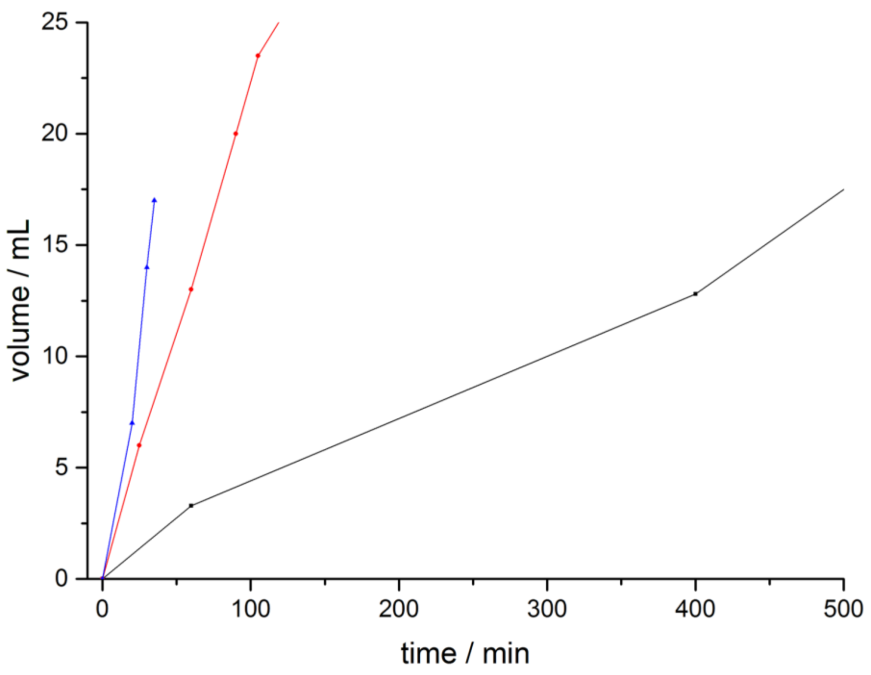

Figure 13. Depolymerization of silicone oil and distillation of cyclic species using **4** as a catalyst. Black: first run, 0.001 mbar, rt. Red: second run, 90 °C, 7 mbar. Blue: third run, 90 °C, 7 mbar.

Table 1. Silanolate salt **[1H][D_3OH]** (**4**) as a catalyst for the depolymerisation of trimethylsilyl endblocked polydimethylsiloxane.

**catalyst**	**molar ratio / mol% [cat./(Si-O)-unit]**	**T / °C**	**p / mbar**	**distillation speed / mLh^{-1}[c]**
**4**[a]	0.1	rt	0.001	3.1(2)
	0.1	90	7	24.7(19)
KOH[1][a]	13	90	7	< 1
KOH[1][b]	2	90	7	0
NaOH[b]	13	90	7	0

[a] Used as solid. [b] Used as aqueous solution. [c] Averaged distillation speed.

Table 2. Composition of the collected volatile compounds of two depolymerisations of polydimethylsiloxane (Roth Siliconöl M 200, Art.-Nr. 4030.1) at room temperature and a pressure of 0.001 mbar employing **[1H][D_3OH]** (**4**) as a catalyst. D_3, D_4 and D_5 mean hexamethylcyclotrisiloxane, octamethylcyclotetrasiloxane and decamethylcyclopentasiloxane.

Silicon compound	Yield / %[a]	Yield / %[a]	Average yield / %
Me_3SiOH / $(Me_3Si)_2O$	11	6	8
D_3	2	2	2
D_4	80	83	82
D_5	7	9	8

[a] Determined via $^{29}Si\{^1H\}$IG NMR spectroscopy.

Table 3. Composition of the collected volatile compounds of two depolymerisations of polydimethylsiloxane (Roth Siliconöl M 200, Art.-Nr. 4030.1) at 90 °C and a pressure of 7 mbar employing **[1H][D_3OH]** (**4**) as a catalyst. D_3, D_4 and D_5 mean hexamethylcyclotrisiloxane, octamethylcyclotetrasiloxane and decamethylcyclopentasiloxane.

Silicon compound	Yield / %[a]	Yield / %[a]	Average yield / %
Me_3SiOH / $(Me_3Si)_2O$	5	6	6
D_3	7	6	6
D_4	79	79	79
D_5	9	9	9

[a] Determined via $^{29}Si\{^1H\}$IG NMR spectroscopy.

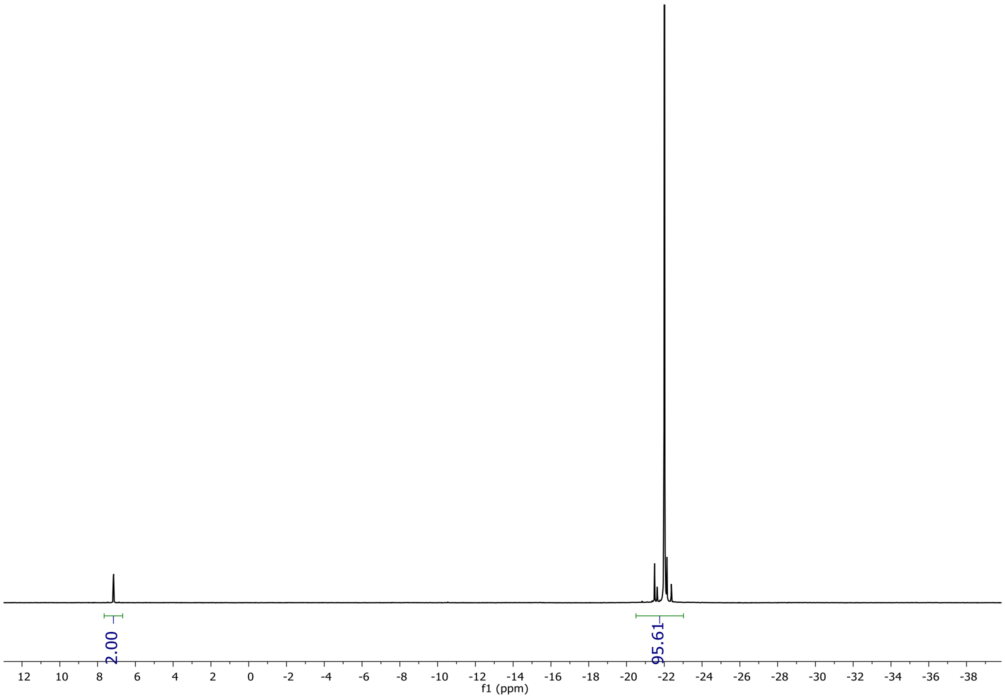

Figure 14. $^{29}Si\{^1H\}$dept30 NMR spectrum of trimethylsilyl endblocked polydimethylsiloxane (Roth Siliconöl M 200, Art.-Nr. 4030.1) in chloroform-d_1 (500 MHz).

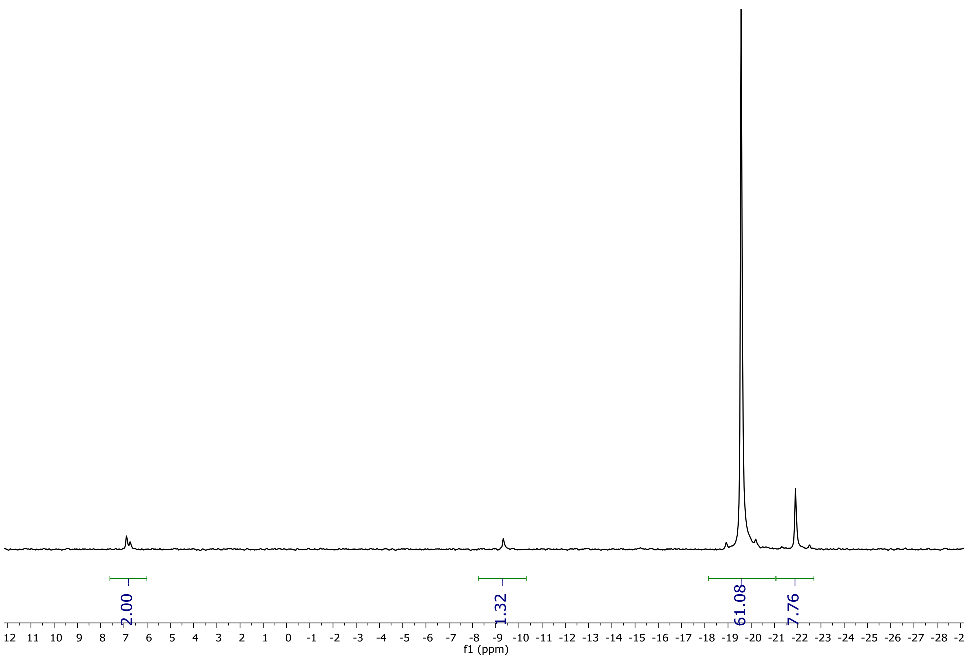

Figure 15. $^{29}Si\{^{1}H\}$IG NMR spectrum of the crude distillate of a depolymerization reaction at room temperature and 0.001 mbar using trimethylsilyl endblocked polydimethylsiloxane and **4** as a catalyst (lock with acetone-d_6 in a capillary) (300 MHz).

1.5 Details on the X-Ray Diffraction

The crystal data were collected on a Rigaku Supernova diffractometer (Cu-K_α radiation (λ = 154.184 pm) at 100.0(2) K).

Using Olex2[2], the structures were solved with the ShelXS[3] structure solution program using direct methods and refined with the ShelXL[4] refinement package using least squares minimization.

In **3** the ratio of disorder of O3A-HA and O3B...HB was refined to 79(2):21(2).

The distances O3A-HA and O1-HB were restrained to be same.

The adp's of O3A and O3B were constrained to be same.

In **4** N13, C35-C38 are disordered in a ratio of 94:6, the minor occupied atoms were restrained using "ISOR 0.001 0.002". The P4-N13 and P4-N13B distances were restrained to be same as well as the distances O1-HA and O4-HB. The ratio of HA:HB was refined to 506(6):494(6). Using a model without this disorder, the U_{eq} value of this hydrogen atom becomes unreasonably large and the R-values increase slightly.

The crystal of **5** was a non-merohedrical twin, with component 2 rotated by 180.0° [0.02 1.00 -0.05] (reciprocal) or [0.01 1.00 -0.04] (direct). Three ethyl groups (C63, C64, C65, C66 and C68) were disordered with a ratio of 78:22. Hydrogen atoms were taken into account using a riding model; only donor hydrogen atoms, i.e. HA, HB and H1, were refined isotropically. HA and HB were disordered, a ratio of 1:1 was assumed. The distances O1-HA and O4-HB were restrained to be same.

Details of the X-ray investigation are given in Table 3. CCDC 1952715 – 1952717 contain the supplementary crystallographic data for this paper. These data can be obtained free of charge via http://www.ccdc.cam.ac.uk/conts/retrieving.html.

Table 4. Structure refinement data of **[1H][Me$_3$SiO(HOSiMe$_3$)$_2$]** (**3**), **[1H][D$_3$OH]** (**4**) and **[1H][D^{Ph2}$_3$OH]** (**5**).

compound	**3**	**4**	**5**
Crystallographic Section			
empirical formula	$C_{49}H_{129}N_{13}O_3P_4Si_3$	$C_{46}H_{119}N_{13}O_4P_4Si_3$	$C_{76}H_{131}N_{13}O_4P_4Si_3$
a / pm	1365.42(2)	1101.915(13)	1663.91(12)
b / pm	1519.23(3)	2358.13(3)	2276.99(7)
c / pm	1715.28(2)	2495.13(3)	2312.88(13)
α / °	91.7566(13)	90	90
β / °	90.5974(12)	93.5860(11)	108.284(7)
γ / °	98.9787(14)	90	90
V / 10^6 pm^3	3512.49(10)	6470.81(13)	8320.4(9)
Z	2	4	4
ρ_{calc} / $mg{\cdot}mm^{-3}$	1.094	1.157	1.197
crystal system	triclinic	monoclinic	monoclinic
space group	*P*-1	$P2_1/c$	$P2_1/n$
color shape	yellowish prisms	colorless needles	colorless prisms
crystal size / mm^{-3}	0.29 × 0.13 × 0.07	0.29 × 0.09 × 0.05	0.33 × 0.30 × 0.17
Data collection			
μ / mm^{-1}	1.827	1.985	1.674
F(000)	1280.0	2480.0	3248
2θ range for data col. / °	5.2 to 151.3°	5.2 to 144.3°	5.6 to 153.0°
index ranges	$-16 \le h \le 15$<br>$-18 \le k \le 18$<br>$-12 \le l \le 21$	$-13 \le h \le 13$<br>$-28 \le k \le 28$<br>$-30 \le l \le 30$	$-20 \le h \le 20$<br>$-24 \le k \le 28$<br>$-28 \le l \le 28$
reflections col.	31246	74329	40620

independent refl.	14332	12607	21342
R(int)	0.0199	0.0321	0.0151
data/restraints/ parameter	14332/0/1169	12607/32/719	21342/13/978
goodness-of-fit on F^2	1.044	1.025	1.063
R_1 / wR_2 [I>2σ(I)]	0.0291/0.0758	0.0339/0.0832	0.0321/0.0875
R_1 / wR_2 (all data)	0.0324/0.0780	0.0430/0.0882	0.0361/0.0894
$\Delta\rho_{max/min}$ / e Å^{-3}	0.41/-0.34	0.34/-0.36	0.46/-0.34
CCDC number	1952715	1952716	1952717

References

[1] A. Oku, W. Huang, Y. Ikeda, *Polymer* **2002**, *43*, 7289.

[2] O. V. Dolomanov, L. J. Bourhis, R. J. Gildea, J. A. K. Howard, H. Puschmann, *J. Appl. Cryst.* **2009**, *42*, 339.

[3] G. M. Sheldrick, *Acta Cryst. A* **2015**, *71*, 3.

[4] G. M. Sheldrick, *Acta Cryst. C* **2015**, *71*, 3.
